# Diagnostic accuracy of contrast-enhanced ultrasound for characterization of kidney lesions in patients with and without chronic kidney disease

**DOI:** 10.1186/s12882-017-0681-8

**Published:** 2017-08-09

**Authors:** Emily Hueywen Chang, Wui Kheong Chong, Sandeep Kumar Kasoji, Julia Rose Fielding, Ersan Altun, Lee B. Mullin, Jung In Kim, Jason Peter Fine, Paul Alexander Dayton, Wendy Kimryn Rathmell

**Affiliations:** 1 University of North Carolina, 7024 Burnett Womack, CB 7155, Chapel Hill, NC 27599 USA; 2 Present address: University of Texas Southwestern at Dallas, 5323 Harry Hines Boulevard, Dallas, TX 75390-8827 USA; 3 Present address: Department of Medicine, Division of Hematology and Oncology, Vanderbilt University, 777 Preston Research Building, Nashville, TN 37232 USA; 4 Diagnostic Radiology, Abdominal Imaging Section, The University of Texas MD Anderson Cancer Center, Unit 1473 FCT15.5092, 1400 Pressler Street, Houston, TX 77030 USA; 5 Department of Radiology, University of North Carolina at Chapel Hill, CB 7510, Chapel Hill, NC 27599 USA; 6 Department of Biostatistics, University of North Carolina, 3101 McGavran-Greenberg Hall, CB #7420, Chapel Hill, NC 27599-7420 USA; 7 Joint Biomedical Engineering Department, University of North Carolina at Chapel Hill/NCSU, CB 7575, Chapel Hill, NC 27599 USA; 8 University of North Carolina, Lineberger Cancer Center, NC 27599, Chapel Hill, USA

**Keywords:** Contrast, Ultrasound, Contrast-enhanced ultrasound, Kidney, Kidney lesion, Chronic kidney disease

## Abstract

**Background:**

Patients with chronic kidney disease are at increased risk of cystic kidney disease that requires imaging monitoring in many cases. However, these same patients often have contraindications to contrast-enhanced computed tomography and magnetic resonance imaging. This study evaluates the accuracy of contrast-enhanced ultrasound (CEUS), which is safe for patients with chronic kidney disease, for the characterization of kidney lesions in patients with and without chronic kidney disease.

**Methods:**

We performed CEUS on 44 patients, both with and without chronic kidney disease, with indeterminate or suspicious kidney lesions (both cystic and solid). Two masked radiologists categorized lesions using CEUS images according to contrast-enhanced ultrasound adapted criteria. CEUS designation was compared to histology or follow-up imaging in cases without available tissue in all patients and the subset with chronic kidney disease to determine sensitivity, specificity and overall accuracy.

**Results:**

Across all patients, CEUS had a sensitivity of 96% (95% CI: 84%, 99%) and specificity of 50% (95% CI: 32%, 68%) for detecting malignancy. Among patients with chronic kidney disease, CEUS sensitivity was 90% (95% CI: 56%, 98%), and specificity was 55% (95% CI: 36%, 73%).

**Conclusions:**

CEUS has high sensitivity for identifying malignancy of kidney lesions. However, because specificity is low, modifications to the classification scheme for contrast-enhanced ultrasound could be considered as a way to improve contrast-enhanced ultrasound specificity and thus overall performance. Due to its sensitivity, among patients with chronic kidney disease or other contrast contraindications, CEUS has potential as an imaging test to rule out malignancy.

**Trial registration:**

This trial was registered in clinicaltrials.gov, NCT01751529.

**Electronic supplementary material:**

The online version of this article (doi:10.1186/s12882-017-0681-8) contains supplementary material, which is available to authorized users.

## Background

The incidence of kidney cancer is increasing with over 60,000 new kidney cancer diagnoses projected for 2017. Kidney cancer is also deadly. Over 14,000 associated deaths are expected in 2017 [1]. If detected early, kidney cancer can be treated effectively with surgery alone. Benign cystic lesions are common, accounting for up to 30% of all identified lesions [2]. Distinguishing malignant kidney neoplasms from benign lesions is crucial to determining appropriate treatment. Diagnostic options include biopsy [3, 4] or interval imaging over months or years [5–7]. Cystic lesions can be characterized with contrast-enhanced computed tomography (CT) or magnetic resonance (MR) imaging [8] using the Bosniak criteria [9, 10].

However, Bosniak classification requires contrast enhancement which may pose risk in some patients with compromised kidney function. Risks associated with iodinated contrast agents used with CT imaging include further impairment in kidney function for those with advanced stages of chronic kidney disease (CKD). Use of gadolinium-based contrast agents in this population also poses a rare but serious risk of nephrogenic sclerosing fibrosis. Incidence of this disease has virtually disappeared with more stringent screening prior to gadolinium exposure and use of agents with better risk profiles. However, gadolinium deposition in various organs is now being postulated even in patients with normal kidney function, although the clinical significance is not yet known [11, 12]. Patients with impaired kidney function experience prolonged exposure to gadolinium since it is cleared primarily by the kidneys and thus have an increased risk of gadolinium deposition [13]. Over 40 million people in the United States have chronic kidney disease (CKD), and this population is rapidly growing (USRDS 2016). These patients have substantially increased risk for the development of kidney cancer [14–16]. However, the typical screening tools of contrast-enhanced CT and MR are often contraindicated in patients with advanced stages of CKD. In patients with moderate stages of CKD, kidney-protective measures can be taken but add additional steps, can be cumbersome and do not entirely eliminate risk. Even in these populations, if a patient has a high-risk lesion, the benefits of a contrast CT or MR study may outweigh the risks from contrast exposure. For patients with lower or moderate risk lesions, the risk of contrast-enhanced CT or MR may not be worth the benefit. Radiologic options for this population are limited [17]. Non-contrasted surveillance imaging is often performed, but this is inferior to contrast enhanced studies for lesion characterization.

A potential alternative imaging modality for evaluating kidney lesions is contrast-enhanced ultrasound (CEUS). CEUS has been used to characterize indeterminate lesions in multiple organs [18, 19], including the kidneys [20–27]. CEUS has the ability to detect vascularization as the microbubble contrast agent remains purely intravascular [28, 29]. A unique aspect of CEUS is its ability to image contrast dynamics in real time [30, 31]. US contrast agents have a low serious adverse event rate of 0.006–0.009%, consisting primarily of anaphylactoid reactions that resolve when the contrast is cleared and typically do not necessitate hospitalization [32, 33]. Less serious side effects that are also rare and typically transient, resolving when the contrast agent is cleared, include headache, dizziness, flushing, nausea, flank pain and chest pain. An additional benefit setting them apart from other contrast agents is that they are excreted by exhalation through the lungs and are therefore not nephrotoxic and safe for patients with kidney disease. Despite the potential for CEUS as a diagnostic option among individuals with CKD, few studies have evaluated CEUS in this population, although interest is increasing [34, 35]. In this exploratory pilot study, we sought to investigate the diagnostic accuracy of CEUS for detecting enhancement of kidney lesions, and thus risk of malignancy, in populations with and without CKD and subsequently determine if this imaging modality has potential to be an alternative screening tool in patients with contra-indications to contrast CT or MR. We hypothesized that CEUS has sensitivity comparable to contrast-enhanced CT or MR among individuals with and without CKD.

## Methods

### Study design and participants

We performed a prospective imaging study of CEUS in patients with kidney lesions identified by prior imaging (ultrasound, CT or MR) obtained as part of routine clinical care. This study was performed in compliance with the policies related to the use of human subjects of the Biomedical Institutional Review Board. All procedures performed were in accordance with the ethical standards of the institutional research committee and with the 1964 Helsinki declaration and its later amendments or comparable ethical standards. Informed consent was obtained from all individual participants included in the study. This pilot study was designed to generate needed data to allow for accurate power calculations in future studies. The numbers of true positives and true negatives were random and not fixed by the study design. The total sample size of 48 was chosen based on based on estimated ability to recruit 2–3 patients a month over a 2-year time period. Any patient meeting inclusion/exclusion criteria was offered participation in the study. Inclusion criteria were: 1) eligibility for nephrectomy or ablative therapy based on identification of a kidney lesion on prior imaging, *or* having CKD and an incompletely characterized kidney lesion on prior imaging; and 2) the ability to provide informed consent and comply with protocol requirements.

Exclusion criteria included: 1) active cardiac disease, including class IV congestive heart failure, unstable angina, severe arrhythmia, myocardial infarction within 14 days prior to the study and uncontrolled blood pressure (>150/90 mmHg); 2) severe pulmonary hypertension or adult respiratory distress syndrome; 3) hypersensitivity to the Definity (Perflutren lipid) US contrast agent; 4) critical illness or intensive care unit status; 5) right-to-left cardiac shunt; 6) unstable neurologic disease within 3 months; 7) invasive kidney procedure between time of lesion identification and CEUS; 8) mental illness or drug abuse; and 9) pregnancy or lactation. Patients were recruited from Urology and Nephrology clinics from July 2013 to November 2014. After obtaining informed written consent, patients underwent CEUS per a standard study protocol with low mechanical index (MI) (0.19) imaging.

Because the Bosniak criteria [9, 10] were not designed for ultrasound, for cystic lesions, we applied an adapted Bosniak criteria to CEUS, including both B-mode and CEUS images, (Additional file 1: Table S1) by substituting “internal echogenicity” within a cyst for “high-attenuation”. High attenuation within a cyst is an indicator of proteinaceous or hemorrhagic content, which appears on US as internal echogenicity. Lesions were categorized as solid or Bosniak I-IV by CEUS. In primary analyses, the CEUS diagnosis was compared to the reference standard of tissue diagnosis (malignant/benign). CEUS Bosniak I, II and IIF lesions were considered negative since these are generally managed non-surgically; CEUS Bosniak III, IV and solid lesions were considered positive since these are generally managed surgically. In secondary analyses, we used a reference standard of tissue diagnosis *or* follow-up imaging (obtained 12–26 months after CEUS). The follow-up interval and imaging modality was determined by the individual’s doctor. Follow-up imaging modalities included standard B-mode US, contrast CT and contrast MR. For patients with more than one follow-up imaging examination, the last available examination was used for analysis. Stable lesions were considered negative. A worsening of concerning lesion characteristics (septations, calcifications, mural thickness, irregularity or nodules) was considered positive. The reference standard of follow-up imaging was used because tissue diagnosis was not feasible in many patients given the risks associated with surgery and biopsy.

In separate analyses among patients who had a clinical contrast-enhanced CT or MR (*n* = 25), CEUS Bosniak classification was compared to CT/MR Bosniak classification, by the same blinded readers, to determine inter-modality agreement. To determine inter-reader agreement, answers to lesion characteristic questions (Additional file 1: Table S1) were compared across readers for CEUS and CT/MR studies. The median time interval between CEUS and CT/MR was 29 days (interquartile range: 20–44).

### Imaging procedure and analysis

CEUS was performed with the Siemens Acuson Sequoia 512 (Siemens, Mountain View, CA, USA) with contrast specific software using a 4C1 abdominal transducer. A standard MI of 1.9 was used for B-mode imaging and a low MI of 0.19 was used for all CEUS clips. The MI is a metric used to describe potential bioeffects caused by ultrasound, specifically cavitation bioeffects. A higher MI indicates increased likelihood of bioeffects. Typical B-mode ultrasound imaging uses an MI of 1.9. Low MI (0.19) is used in CEUS to avoid disruption of microbubbles. Scanning was performed by registered sonographers trained in contrast imaging. Lesions were located with B-mode ultrasound. For patients with multiple lesions, the most complex lesion as designated on prior imaging was chosen for CEUS imaging. The transducer was then positioned over the lesion so that the imaging plane included part of the normal kidney parenchyma. The contrast agent, Perflutren Lipid microspheres (Definity^R^, Lantheus, North Billerica, MA), was prepared as a bolus injection, as described in the package insert instructions. The total contrast volume administered was based on weight, 0.50 mL for <125 lb., 0.65 mL for 125–185 lb., and 1.0 mL for >185 lb. This contrast was diluted in saline to a final volume of 5 mL and injected over 15 s, followed by a 5 mL saline flush. Lesions were imaged for 3 min after contrast injection.

Images were de-identified and interpreted by two radiologists blinded to lesion diagnosis and to each other’s reads. A custom graphical user interface (GUI) developed in MATLAB (MathWorks, Natick, MA) displayed de-identified CEUS and B-mode clips accompanied by questions related to lesion characteristics (Additional file 2: Figure S1). Two abdominal radiologists (identified as Reader 1 and Reader 2 with 20 and 15 years’ experience in ultrasound imaging, respectively) not involved in image acquisition independently performed the blinded study reads. Both readers participated in a preliminary training session and performed CEUS radiology literature review, particularly from experienced centers such as the University of Calgary, and international ultrasound societies [36]. Readers used both B-mode and CEUS images to answer lesion characteristic questions, classify the lesions as solid or cystic, and if cystic, apply the adapted Bosniak criteria (Additional file 1: Table S1). CEUS enhancement was determined by the subjective determination of the appearance of contrast on the image. The diagnosis based on the B-mode and CEUS images is designated “CEUS diagnosis”. The same radiologists reviewed the initial contrast-enhanced CT and MR images when available and answered the same questions. A 3-month delay between CEUS and CT/ MR readings was instituted to prevent recall.

Contrast-enhanced CTs were acquired with a 16- to 64-slice MDCT scanner. A non-contrasted scan was first obtained. 100 mL of Iohexol 755 mg/ml (Omnipaque 350 – GE Healthcare, Milwaukee, WI) was administered intravenously at 3-4 mL/s. MRs were performed on 1.5 Avanto and 3 T Trio (Siemens, Iselin, NJ, USA). 10 ml of Gadobenate Diglumine (Multihance, Bracco Diagnostics, Monroe Township, NJ) was administered intravenously. For details about the CEUS, CT and MR imaging techniques, see Additional file 3.

### Statistical analysis

For the primary analysis, CEUS diagnosis was compared to the reference standard of tissue diagnosis. For secondary analyses, CEUS diagnosis was compared to tissue diagnosis *or* follow-up imaging diagnosis. Sensitivity, specificity and overall accuracy (number of CEUS correct diagnoses/total number of lesions) were calculated for each reader separately, with 95% confidence intervals using the exact Pearson-Clopper method. Reader data were considered separately and combined, with measures calculated using generalized estimating equations [37], along with asymptotic 95% confidence intervals under a working independence assumption between readers.

Performance measures (sensitivity, specificity and overall accuracy) were also determined for the subset of patients with CKD, with analyses analogous to those used in the full cohort. These exploratory secondary analyses were stratified according to CKD severity: early CKD (GFR ≥ 30 mL/min) and advanced CKD (GFR < 30 mL/min, including patients on dialysis or the native kidney of a patient with a kidney transplant). Because of small sample sizes, the current study was underpowered to demonstrate statistically significant differences in diagnostic accuracy between late and early CKD groups. To detect the observed differences in total accuracy of 0.8 versus 0.5 at significance level 0.05 using a 2-sided test of two proportions, 40 subjects in each CKD group would be needed for 80% power and 50 subjects per group for 90% power.

Additional exploratory subgroup secondary analyses considered accuracy across patients with a priori-designated co-morbid conditions (hypertension, diabetes mellitus, cardiovascular disease and obesity) and across lesion sizes (<3 vs. ≥3 cm). Fisher’s exact test was used to calculate statistical significance. Agreement between readers was calculated using sample proportions. All analyses were conducted in SAS version 9.4 (Cary, NC). Two-tailed *p*-values of <0.05 were used to indicate statistical significance.

## Results

### Patient and lesion characteristics

**Fig. 1:**
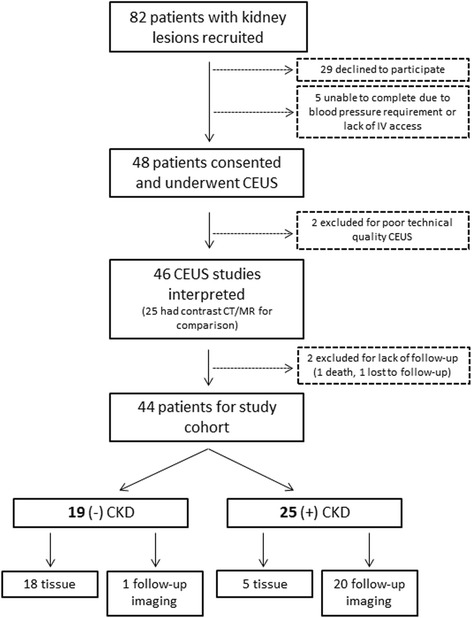
Patient flowchart. Flowchart of patient recruitment, exclusions and numbers for final analysis

A flowchart of patient recruitment is shown in Fig. 1. Of the 48 patients who underwent CEUS examination, 2 were excluded due to poor technical quality. Of the 46 patients that received CEUS classification, 2 were excluded due to lack of follow-up. One died of unrelated cause (meningitis), and another patient with a suspicious lesion recommended for nephrectomy opted for surveillance, but was lost to follow-up. A total of 44 patients had interpretable CEUS imaging with one lesion examined per patient and either tissue diagnosis or follow-up imaging results available. A summary of patient and lesion characteristics is displayed in Table 1. Mean patient age was 56, with 70% (31/44) being male. The most common co-morbidities were hypertension (68%, 30/44), hyperlipidemia (50%, 22/44) and obesity (50%, 22/44). Of the 44 patients, 25 had CKD, including 7 patients receiving dialysis and 3 who had received a kidney transplant. All 3 had functioning transplants. Patients with CKD were more likely to be older and have hypertension and cardiovascular disease.

**Table 1:** Patient and lesion characteristics

	Total (*N* = 44)	(+) CKD (*n* = 25)	(−) CKD (*n* = 19)
Age (years)			
Mean ± S.D.	56 ± 14	59 ± 13	51 ± 14
Male	31 (70%)	18 (72%)	13 (68%)
Co-morbid conditions			
Hypertension	30 (68%)	20 (80%)	10 (53%)
Diabetes	10 (23%)	5 (20%)	5 (26%)
Hyperlipidemia	22 (50%)	13 (52%)	9 (47%)
Cardiovascular disease	9 (20%)	8 (32%)	1 (5%)
History of renal cell carcinoma	3 (7%)	2 (8%)	1 (5%)
Obesity^a^	22 (50%)	11 (44%)	11 (58%)
Initial Study^b^			
Non-contrast CT	11 (25%)	8 (32%)	3 (16%)
Contrast CT	20 (45%)	5 (20%)	15 (79%)
Non-contrast MR	1 (2%)	0	1 (5%)
Contrast MR	6 (14%)	1 (4%)	5 (26%)
Conventional US	17 (39%)	17 (68%)	0
Laterality of lesion			
Right	19 (43%)	12 (48%)	7 (37%)
Left	20 (45%)	9 (36%)	11 (58%)
Bilateral	5 (11%)	4 (16%)	1 (5%)
Laterality of imaging			
Right	21 (48%)	14 (56%)	7 (37%)
Left	23 (52%)	11 (44%)	12 (63%)
Diameter by imaging (cm)^c^			
Mean (range)	3.27 (1.4–7.9)	3.09 (1.4–7.9)	3.51 (1.4–6.6)
Diameter by histology (cm)^d^			
Mean (range)	3.39 (0.4–7) (*n* = 20)	2.88 (0.4–5.6) (*n* = 4)	3.51 (1.2–7) (*n* = 16)
Diagnosis			
Clear cell RCC	14 (32%)	1 (4%)	13 (68%)
Papillary RCC	5 (11%)	3 (12%)	2 (11%)
Chromophobe RCC	2 (4%)	1 (4%)	1 (5%)
Angiomyolipoma	1 (2%)	0	1 (5%)
Oncocytoma	1 (2%)	0	1 (5%)
Surveillance	21 (48%)	20 (80%)	1 (5%)
Stable/benign	19	19	0
Progressed/malignant	2	1	1
CKD stage			
Non-CKD	19 (43%)	0	19 (100%)
CKD II	2 (4%)	2 (8%)	0
CKD III	9 (20%)	9 (36%)	0
CKD IV	4 (9%)	4 (16%)	0
CKD V on dialysis	7 (16%)	7 (28%)	0
Transplant	3 (7%)	3 (12%)	0
Probable cause of CKD			
Hypertensive Nephrosclerosis		11 (44%)	
Diabetic Kidney Disease		4 (16%)	
Hypertension/Diabetic Kidney Disease		1 (4%)	
Polycystic Kidney Disease		2 (8%)	
Chronic Interstitial Nephritis		2 (8%)	
Membranous Nephropathy		1 (4%)	
Focal Segmental Glomerulosclerosis		1 (4%)	
Medullary Sponge Kidney		1 (4%)	
NSAID-induced Nephropathy		1 (4%)	
Lithium Nephrotoxicity		1 (4%)	

*CKD* chronic kidney disease, *CT* computed tomography, *MR* magnetic resonance imaging, *US* ultrasound, *RCC* renal cell carcinoma

^a^Defined as BMI ≥ 30

^b^Percentages add to greater than 100% as 11 patients had multiple studies prior to CEUS

^c^By largest dimension

^d^One sample was resected in multiple pieces with no histologic diameter available

The most common initial study for patients without CKD was contrast CT (*n* = 15) followed by contrast MR (*n* = 5). The most common initial study for patients with CKD was a conventional, non-contrasted ultrasound (*n* = 17), followed by non-contrasted CT (*n* = 8). Patients with CKD were more likely to have bilateral lesions with smaller diameters of individual lesions. The average diameter based on initial imaging study was 3.51 cm in non-CKD patients and 3.09 cm in CKD patients.

Of the 44 patients in the study cohort (Fig. 1), 23 underwent surgery/biopsy and 21 were followed with serial imaging. Of the 23 surgical patients, 22 had nephrectomy and 1 had fine needle aspiration showing angiomyolipoma. The primary reference standard of histologic diagnosis categorized 2 lesions as benign and 21 as malignant. The observed malignancy subtypes (clear cell, papillary, and chromophobe) followed patterns observed in prior studies [38]. More papillary renal cell carcinomas were observed in patients with CKD compared to patients without CKD, as has been previously observed [39]. The secondary reference standard of tissue diagnosis or diagnosis on follow-up imaging categorized 21 lesions as benign (2 by histology and 19 by imaging) and 23 as malignant (21 by histology and 2 by imaging). The mean follow-up period was 20 months and ranged from 12 to 26 months.

### CEUS accuracy

Accuracy was defined as the number of CEUS correct diagnoses/total number of lesions. Table 2 displays combined and individual reader accuracy results, including sensitivity, specificity and overall accuracy for the diagnosis of kidney cancer. In the primary analysis using tissue diagnosis as the reference standard, combined overall accuracy was 87% (95% CI: 69%, 95%). In secondary analyses using tissue or follow-up imaging as the reference standard, combined overall accuracy was 73% (95% CI: 59%, 83%). The cohort size was inadequate for analyzing CEUS characterization based on histological subtype (clear cell, papillary, or chromophobe).

**Table 2:** Accuracy of CEUS compared to tissue diagnosis (primary analysis) and tissue diagnosis or follow-up imaging diagnosis (secondary analysis)^a^

	Reader 1		Reader 2		Combined readers	
	Tissue diagnosis (*N* = 23)	Tissue or follow-up imaging (*N* = 44)	Tissue diagnosis (*N* = 23)	Tissue or follow-up imaging (*N* = 44)	Tissue diagnosis (*N* = 23)	Tissue or follow-up imaging (*N* = 44)
Sensitivity	90% (78%, 100%) [19/21]	91% (80%, 100%) [20/22]	100% (100%, 100%) [21/21]	100% (100%, 100%) [22/22]	95% (83%, 99%)	96% (84%, 99%)
Specificity	0% (0%, 0%) [0/2]	59% (39%, 80%) [13/22]	0% (0%, 0%) [0/2]	41% (20%, 61%) [9/22]	0% (0%, 0%)	50% (32%, 68%)
Overall Accuracy	83% (67%, 98%) [19/23]	75% (62%, 88%) [33/44]	91% (80%, 100%) [21/23]	70% (57%, 84%) [31/44]	87% (69%, 95%)	73% (59%, 83%)

^a^Results presented as accuracy metrics (95% CI) and [positive or negative reads/total reads]. CEUS images were read by 2 independent readers. Results were considered per reader and as a combination of the 2 readers. For combined reader result, generalized estimating equations were used considering two readers’ values as repeated measurements (or responses)

### Inter-reader agreement

**Fig. 2:**
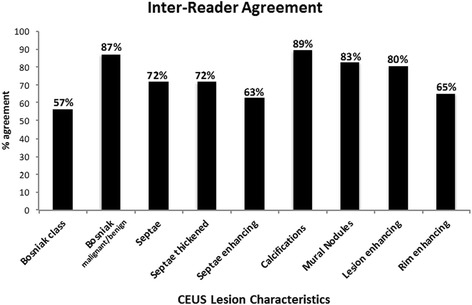
Inter-reader agreement. The rate of agreement between the two readers was calculated for each individual lesion characteristic, the Bosniak class designation (or solid designation) and the overall designation of malignant or benign based on Bosniak class (Bosniak I, II and IIF considered benign and Bosniak III, IV and solid considered malignant)

CEUS inter-reader agreement results are shown in Fig. 2. The highest rate of agreement was seen with calcifications (89%), overall malignant/benign designation (87%), mural nodules (83%) and lesion enhancement (80%). The lowest rate of agreement was with Bosniak class (57%), septa (63%) and rim enhancement (65%). Low rate of Bosniak class agreement was not surprising as there are 6 possible categories (solid, I, II, IIF, III and IV).

### CEUS diagnoses compared to contrast CT/MR diagnoses

CEUS diagnosis was compared to CT/MR diagnosis for 25 patients who had both CEUS and a contrasted CT/MR. Of the studies that were positive (Bosniak III, IV or solid) by CT/MR, 89% were also positive by CEUS for reader 1 and 96% for reader 2 (Table 3). Of the studies that were negative (Bosniak I, II and IIF) by CT/MR, 83% were read as positive by CEUS for reader 1 and 100% for reader 2. There was no overlap in confidence intervals for these two groups for either reader. Overall agreement between CT/MR and CEUS diagnoses was 72% for reader 1 and 92% for reader 2. Inter-reader agreement with CT/MR was found to be 81% for malignant/benign designation.

**Table 3:** Agreement between contrast CT/MR and CEUS diagnosis^a^

		Reader 1	Reader 2
CT/MR+	CEUS+	89% (76%, 100%) [17/19]	96% (87%, 100%) [23/24]
	CEUS-	11% (0%, 24%) [2/19]	4% (0%, 13%) [1/24]
CT/MR-	CEUS+	83% (54%, 100%) [5/6]	100% (100%, 100%) [1/1]
	CEUS-	17% (0%, 46%) [1/6]	0% (0%, 0%) [0/1]
	Overall Agreement	72% (54%, 90%) [18/25]	92% (81%, 100%) [23/25]

^a^Results presented as rate of agreement with (95% CI) and [CEUS interpretation/CT or MR interpretation]

### Accuracy in patients with chronic kidney disease

Of the 25 patients with CKD, 5 (20%) had tissue diagnoses. All 5 of these patients had malignant lesions, and sensitivity of CEUS in these patients was 90% (95% CI: 56%, 98%). Compared to tissue or follow-up diagnosis, sensitivity was 90% (95% CI: 56%, 98%) and specificity 55% (95% CI: 36%, 73%). Overall accuracy was 62% (95% CI: 44%, 77%). Further analysis by CKD stage showed CEUS sensitivity, specificity and overall accuracy was lower in patients with advanced (stage IV, V, dialysis and transplant) versus early (stage II and III) CKD. The combined overall accuracy for early CKD was 77% (95% CI: 48%, 93%), and 50% (95% CI: 29%, 71%) for advanced CKD. Sensitivity and specificity and individual accuracies for each reader are presented in Table 4.

**Table 4:** Accuracy of CEUS based on severity of CKD compared to the secondary reference standard^a^

	Reader 1		Reader 2		Combined readers	
	Early CKD (eGFR ≥30 mL/min) (*N* = 11)	Advanced CKD (eGFR <30 mL/min) (*N* = 14)	Early CKD (eGFR ≥30 mL/min) (*N* = 11)	Advanced CKD (eGFR <30 mL/min) (*N* = 14)	Early CKD (eGFR ≥30 mL/min) (*N* = 11)	Advanced CKD (eGFR <30 mL/min) (*N* = 14)
Sensitivity	100% (100%, 100%) [2/2]	67% (13%, 100%) [2/3]	100% (100%, 100%) [2/2]	100% (100%, 100%) [3/3]	100% (100%, 100%)	83% (42%, 97%)
Specificity	78% (20%, 100%) [7/9]	55% (25%, 84%) [6/11]	67% (36%, 97%) [6/9]	27% (1%, 54%) [3/11]	72% (40%, 91%)	41% (20%, 66%)
Overall Accuracy (no. correct/total)	82% (59%, 100%) [9/11]	57% (31%, 83%) [8/14]	73% (46%, 99%) [8/11]	43% (17%, 69%) [6/14]	77% (48%, 93%)	50% (29%, 71%)

^a^Results presented as accuracy metrics (95% CI) and [positive or negative reads/total reads]. CEUS images were read by 2 independent readers. Results were considered per reader and as a combination of the 2 readers. For combined reader result, generalized estimating equations were used considering two readers’ values as repeated measurements (or responses)

Abbreviations: CKD, chronic kidney disease; eGFR, estimated glomerular filtration rate

Numbers were not sufficient to detect significant differences in accuracy between patients with and without hypertension, diabetes, cardiovascular disease and obesity (Additional file 1: Table S2) or to perform analyses on subgroups of solid and cystic lesions (Additional file 1: Table S3). Such analyses were considered exploratory and are presented in Additional file 1: Tables S2 and S3.

### Accuracy in patients with chronic kidney disease for small (<3 cm) vs. large (≥3 cm) lesions

Accuracy was examined for lesions <3 cm (*n* = 23) and ≥3 cm (*n* = 21) in diameter in patients with and without CKD. More patients with CKD had smaller lesions (16/23) than larger lesions (9/21). For smaller lesions, when combining results from both readers, overall accuracy decreased from 71% (95% CI: 33%, 93%) for patients with no CKD to 56% (95% CI: 34%, 76%) for patients with CKD. For larger lesions, when combining results from both readers, overall accuracy decreased from 96% (95% CI: 76%, 99%) for patients with no CKD to 72% (95% CI: 46%, 89%) for patients with CKD. There was no difference in sensitivity for larger lesions but a decrease in sensitivity from 100% (95% CI: 100%, 100%) to 75% (95% CI: 32%, 95%) was obersved in smaller lesions. Results are presented in Additional file 1: Table S4.

## Discussion

**Fig. 3:**
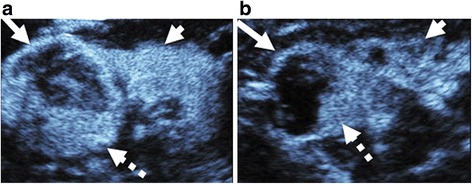
Complex cystic lesions in 2 patients with chronic kidney disease of different severity. Sagittal CEUS images of two patients with complex cystic lesions, called Bosniak IV by both readers (long arrows). Mural nodularity (dashed arrows) is present in both cysts. Both lesions were confirmed as RCC on pathology. However, the 72-year old woman in 3**a** had early kidney disease, and the non-neoplastic kidney parenchyma (short arrow) in this case demonstrates homogeneous enhancement. 3**b** is a case of a 67-year old man with advanced kidney disease on dialysis in which the kidney parenchyma appears very different, demonstrating reduced, patchy enhancement (short arrow)

Evaluation of kidney lesions by ultrasound has historically been limited by lack of enhancement. Contrast-enhanced CT/MR has been the imaging standard. However, iodinated contrast agents may worsen kidney function, and gadolinium agents may cause nephrogenic systemic fibrosis in patients with advanced CKD. The primary goal of this pilot study was to explore the detection of microbubble contrast in kidney lesions among patients with and without CKD and to test the accuracy of CEUS for characterizing these lesions as malignant or benign (Fig. 3). We found that the sensitivity of CEUS, whether compared to tissue diagnosis or follow-up imaging, is high and comparable to the reported sensitivities of CT (83–100%) and MR (81–100%) [21–24, 40] among patients with and without CKD. High sensitivity provides high certainty that a negative test in an individual means the individual does not have the disease, essentially ruling out malignancy.

Although we found high sensitivity with CEUS, specificity was low. Whether compared to tissue diagnosis or tissue diagnosis/follow-up imaging, specificity of CEUS is lower than reported CT (51–96%) and MR (71–100%) specificities [21–24, 40]. Low specificity means a large number of false positives, thus a positive test does not mean the individual has the disease and cannot rule in malignancy very well.

A test with high sensitivity and lower specificity (for example, mammography or CT for lung cancer) is suitable for screening purposes. Benign cystic masses are common in renal insufficiency and distinguishing malignant from benign cysts is a diagnostic challenge. CT or MR is the current standard, but performing CT/MR on all patients with cystic masses would be prohibitively costly and expose a large number of patients to radiation or potentially nephrotoxic contrast agents. CEUS’s ability to exclude malignancy with minimal risk of adverse effects means that the use of CT/MR could be narrowed down to only those patients with a positive CEUS.

**Fig. 4:**
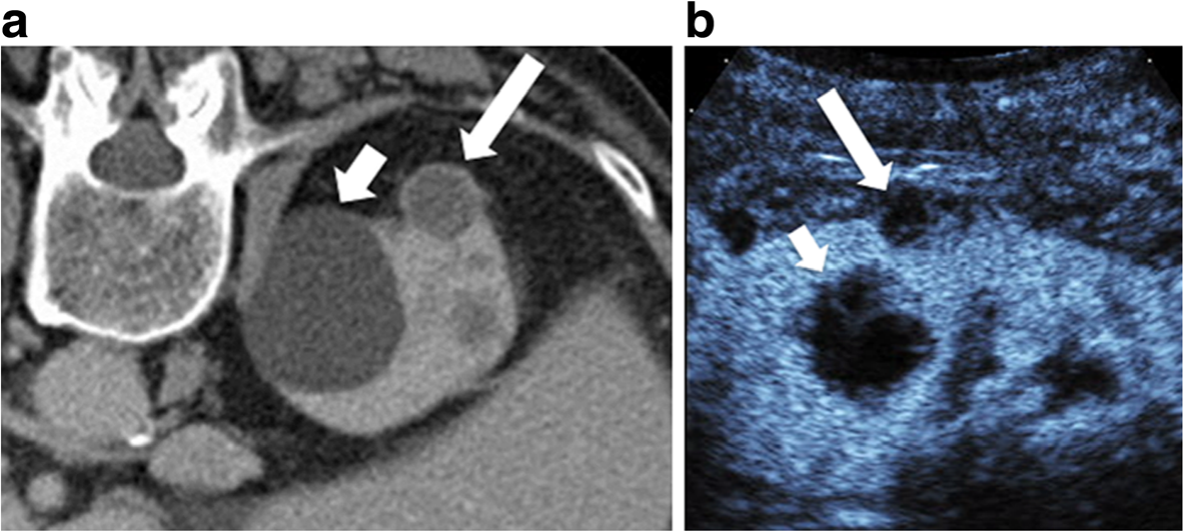
Upstaging of cystic lesion on contrast-enhanced CT and CEUS due to greater special resolution. Contrast enhanced CT (4**a**) showing a smaller hyperdense cyst (long arrow) classified by readers as Bosniak II by one reader and IIF by the other in a 75 year old man. Adjacent to this is a large simple cyst (Bosniak I) (short arrow). On CEUS (4**b**), the smaller cyst (long arrow) shows enhancing internal septa and a solid component invisible on the CT that resulted in a Bosniak III classification. The larger cyst (short arrow) demonstrates internal features such as septations and wall irregularity that were also not visible on the CT. This illustrates the greater spatial resolution of CEUS compared to CT, and may explain why applying the Bosniak criteria to CEUS leads to upstaging

**Fig. 5:**
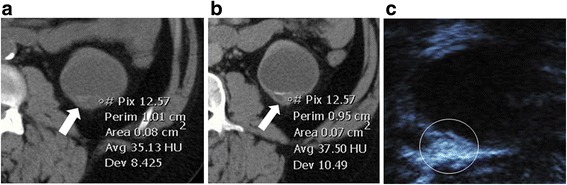
Upstaging of cystic lesion on contrast-enhanced CT and CEUS due to greater contrast resolution. On the contrast-enhanced CT of a 44-year old patient with advanced kidney disease and kidney transplant, wall thickening is present (arrow). ROI shows no enhancement: the same Hounsfield unit measurements were seen pre- (5**a**, 35HU) and post-contrast (5**b**, 37.5 HU). This was read by one radiologist as Bosniak II and the other as Bosniak III. On CEUS (5**c**), the thickened wall is irregular and clearly enhances. Both readers read the lesion (circled) one stage higher as Bosniak III and IV on CEUS. This illustrates that CEUS has greater contrast resolution than CT, and may explain why applying the Bosniak criteria to CEUS leads to upstaging

In the subgroup of patients who also had CT/MR, the number of patients who were CEUS negative but CT/MR positive is smaller than the CEUS positive but CT/MR negative group, suggesting that CEUS tends to assign a higher Bosniak category (or solid designation) than CT/MR (Fig. 4 and Fig. 5). This analysis does not take diagnosis into account and can therefore only be used to suggest that CEUS is more likely to call a lesion malignant than CT/MR and does not lead to conclusions about false positive or negative rates.

There are three possible explanations: First, ultrasound is more sensitive than CT/MR for detecting the presence of intravascular contrast. Unlike CT and MR agents, microbubble contrast agents remain intravascular: they are not filtered by glomeruli and do not extravasate into the interstitial space [41]. This may lead to more prominent internal enhancement with CEUS than CT/MR agents [22, 23]. Second, ultrasound has greater spatial resolution than CT/MR: in some cases (Fig. 4), cysts that appear simple on CT/MR show internal features on ultrasound, including septations not seen on CT, resulting in a higher Bosniak grade using definitions based on CT or MR imaging. Better spatial and contrast resolution is an important advantage to examining lesions with CEUS; however, to reduce the false positive rate, the grading system will need to be modified to take this into account. Future, larger studies should be performed to develop a revised classification system [42]. Using objective measures of enhancement, similar to Hounsfield units for CT, may also improve CEUS accuracy. Furthermore, new CEUS imaging techniques such as super-resolution imaging [43], molecular imaging [44], and acoustic angiography [45] may further improve specificity to malignant lesions as they are translated into the clinic. The third possibility is that relative inexperience with CEUS compared to CT or MR leads to upstaging of kidney lesions.

**Fig. 6:**
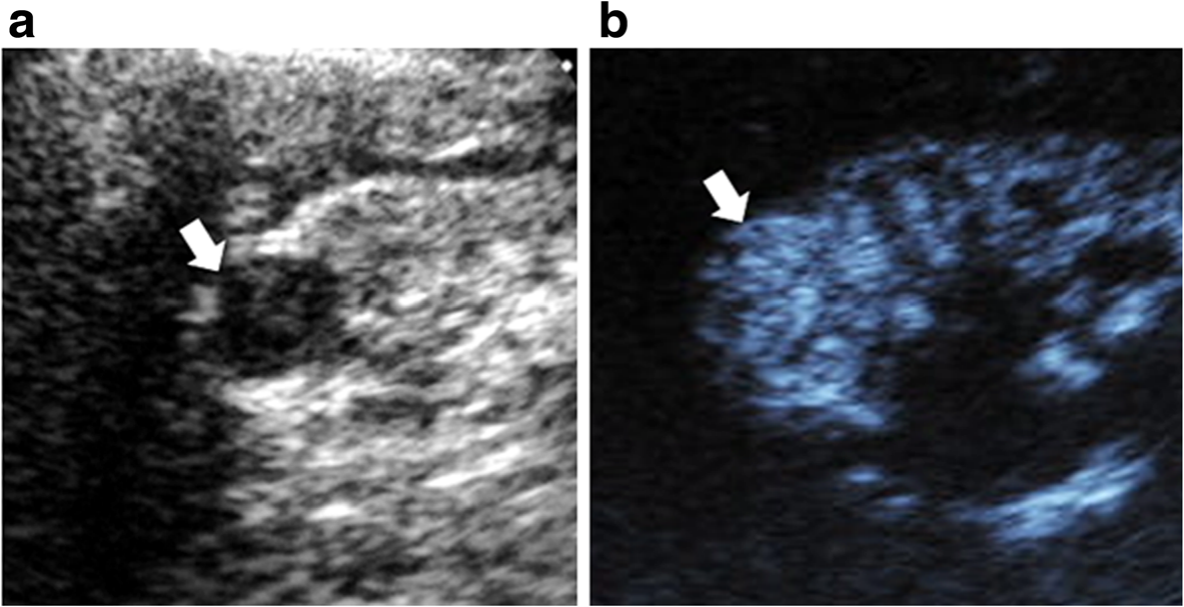
Partly cystic lesion on CEUS compared to gray scale ultrasound. Gray scale longitudinal ultrasound (6**a**) shows an apparently partly cystic lesion within a strongly echogenic kidney in a 53-year old man with advanced CKD and hence contraindications to both contrast CT and MR. The lesion demonstrates intense homogeneous enhancement on CEUS (6**b**) and is larger but otherwise unchanged on 21-month follow-up imaging. It appeared partly cystic on grayscale ultrasound because it was surrounded by strongly echogenic CKD tissue. This case illustrates the difficulty of differentiating tumors from benign cysts on conventional ultrasound in the setting of advanced CKD

Few studies have looked specifically at the use of CEUS for characterizing lesions in patients with CKD [46]. In this study, we examined patients with and without CKD. With more advanced CKD, overall accuracy declined, due largely to a decrease in specificity. While sensitivity only dropped from 100% for early CKD to 83% for advanced CKD, specificity dropped from 72% to 41%. The reason for this requires further investigation, but we observed that while we were able to detect contrast enhancement in the uninvolved parenchyma for all patients, parenchymal enhancement was more heterogeneous and reduced in patients with CKD compared to non-CKD patients. We therefore hypothesize that a decrease in background parenchymal enhancement accentuates the difference in lesion enhancement compared to surrounding parenchyma. This abnormal parenchymal enhancement is even further pronounced in advanced CKD and may cause the lesion to stand out more, creating the perception that it is enhancing. This would be expected to primarily affect specificity, as the false-positive rate would increase with worsening CKD. With this in mind, we hypothesized that subjects with vascular CKD pathology (diabetes or hypertension) would have more false positives than non-vascular etiologies (cystic, glomerular, drug-related) but found no significant differences, likely due to small numbers. This should be explored further in larger populations. Nonetheless, the high sensitivity for ruling out malignancy in the CKD population suggests that this modality could be used for screening following non-contrast ultrasound or CT for patients in whom contrast-enhanced CT/MR is contraindicated (Fig. 6). Larger studies are needed to confirm and extend our findings.

Based on our findings, we suggest that CEUS be considered to evaluate low to moderate-risk lesions in patients who have absolute or relative contra-indications to contrast CT or MR. If CEUS does not detect lesion enhancement, the patient may not need to undergo contrast CT or MR. If CEUS does detect lesion enhancement, further imaging should be considered. In addition, CEUS may have an application as a secondary test for lesions not definitively diagnosed by either contrast CT or MR. Due to the greater sensitivity to enhancement and special resolution, CEUS may be able to detect lesion enhancement where other modalities may not. This potential application should be evaluated in future studies.

Although CEUS is not yet FDA-approved for kidney imaging in the United States, it is increasingly used off label. Interpretation tools, such as the customized GUI, that address each component lesion characteristic of the Bosniak classification system, can assess reader consistency and inter-reader agreement. Observational training visits to institutions that have experience and familiarity with CEUS will improve implementation and interpretation accuracy.

This study has limitations. First, this is a pilot study with a relative small size population from a single center. Due to the small size, meaningful subgroup analyses, such as those at each stage of CKD and those with cystic vs. solid lesions, was not possible. Because of this limitation, the conclusions drawn are preliminary in nature and should be interpreted with caution. Future, larger, multicenter studies are needed to determine the true accuracy of CEUS among individuals with diverse stages of CKD. Second, the blinded readers in this study were academic genitourinary radiologists with fellowship training in ultrasound. They underwent a preliminary training session and performed CEUS literature review, but neither had prior experience with CEUS. However, their background is not atypical as physicians are now becoming more familiar with CEUS in the United States, particularly in academic centers. Reassuringly, the sensitivities observed were similar to those reported by more experienced centers [34]. As there is little available formal training, development of a training program and experience with interpretation of CEUS is needed before widespread implementation. Additionally, because CEUS is a perfusion study, applying objective perfusion parameters could increase accuracy. Third, the reference standard of tissue diagnosis was not available for all patients because many indeterminate lesions underwent imaging surveillance rather than biopsy or surgery. A minimum of one-year follow-up was accepted, but the ideal follow-up period is 3–5 years, depending on lesion complexity, as some renal cancers are slow growing. Until longer follow-up data on more patients is obtained, malignancy cannot be ruled out with a Bosniak IIF lesion, and these patients would still need to be followed, as is the case with contrast CT and MR. Fourth, both cystic and solid lesions were included. In order to more accurately determine accuracy and potential modifications to Bosniak criteria for CEUS in a CKD population, future studies will need to include primarily cystic lesions. Fifth, the referral source for the two groups, non-CKD and CKD, was different. This introduces potential bias since the non-CKD group came primarily from urology clinic where patients were referred for evaluation of highly suspicious lesions (i.e. likely higher risk lesions), and the CKD group came primarily from non-urologists who were longitudinally following indeterminate lesions (i.e. likely lower risk lesions). Lastly, as with any ultrasound study, obesity, lesion location and motion artifact from breathing are technical limitations.

## Conclusion

The traditional Bosniak classification system was developed to classify kidney cystic lesions based on contrast-enhanced CT characteristics We adapted this classification system for B-mode US/CEUS and found that it has potential as a test to exclude malignancy based on a sensitivity comparable to CT/MRI, but it does not yet have good specificity. Low specificity might be attributed to the greater spatial resolution and contrast detection with CEUS compared to contrast-enhanced CT, leading to upstaging of lesions. It may also be attributed to lack of experience interpreting CEUS studies. Our results suggest that with further refinement of a classification system and more training**,** CEUS may be an alternative to contrast CT or MR for characterizing indeterminate kidney lesions, particularly in patients with CKD or other contraindications to contrast CT or MR. However, if the traditional Bosniak criteria are used for CEUS, malignancy can still be excluded but a positive CEUS result will require further diagnostic evaluation due to its low specificity. Improved specificity may be accomplished with training programs for radiologists and revisions to the Bosniak criteria, potentially adopting a classification scheme proposed by Barr et al. [24] which incorporates both solid and cystic lesions. Larger studies focused on cystic lesions, particularly in patients with later stages of CKD are needed to confirm and extend our findings and inform classification criteria revisions.

## Additional files

Additional file 1: Table S1. Bosniak criteria adapted to CEUS. Adaptations in bold. **Table S2.** Overall accuracy of CEUS lesion designation by co-morbid conditions in patients with CKD. **Table S3.** Accuracy of CEUS in patients with purely solid lesions and cystic lesions or lesions with a cystic component. **Table S4.** Accuracy of CEUS compared to tissue diagnosis or follow-up imaging in patients with lesions <3 cm and ≥3 cm in patients with and without CKD. (DOCX 15 kb)

Additional file 2: Figure S1. The custom designed graphical user interface used by the readers to interpret CEUS images (PNG 40123 kb)

Additional file 3: Imaging parameters. (DOCX 13 kb)
