# Supplementary material for: Diagnostic accuracy of contrast-enhanced ultrasound for characterization of kidney lesions in patients with and without chronic kidney disease
Source: BMC Nephrol. 2017 Aug 9;18:266. doi: 10.1186/s12882-017-0681-8 (PMC5551034; doi:10.1186/s12882-017-0681-8)
Supplement: Supplementary file 1 — Bosniak criteria adapted to CEUS. Adaptations in bold. Table S2. Overall accuracy of CEUS lesion designation by co-morbid conditions in patients with CKD. Table S3. Accuracy of CEUS in patients with purely solid lesions and cystic lesions or lesions with a cystic component. Table S4. Accuracy of CEUS compared to tissue diagnosis or follow-up imaging in patients with lesions <3 cm and ≥3 cm in patients with and without CKD. (DOCX 15 kb) [file 12882_2017_681_MOESM1_ESM.docx]

**Table S1** Bosniak criteria adapted to CEUS. Adaptations in bold.

| **Category** | **Ultrasound findings** |
| --- | --- |
| **I** | Cyst with a hairline-thin wall that does not contain septa, calcifications (defined as hyperechoic areas with shadowing) or solid components. It has no **internal echoes**, and does not enhance. |
| **II** | Cyst that may contain a few hairline-thin septa. Fine calcification or a short segment of slightly thickened calcification may be present in the wall or septa.  Cystic lesions with uniform **internal echogenicity** (< 3 cm) that are sharply marginated and do not enhance are included in this group. |
| **IIF** | Cyst may contain an increased number of hairline-thin septa, with possible minimal enhancement or thickening of the septa or wall. The cyst may contain calcification that may be thick and nodular, but there are no enhancing soft-tissue components. Totally intrarenal non-enhancing lesions with uniform **internal echogenicity** that are 3 cm or larger are also included. |
| **III** | These lesions are indeterminate cystic masses that have thickened, irregular walls or septa in which enhancement can be seen. |
| **IV** | These lesions are clearly malignant cystic masses that not only have all the characteristics of category 3 lesions, but also contain enhancing soft-tissue components adjacent to but independent of the wall or septa. |

**Table S2** Overall accuracy of CEUS lesion designation by co-morbid conditions in patients with CKD

|  | N | Combined readers  % correct | p-value^a^ |
| --- | --- | --- | --- |
| (+) Hypertension  (-) Hypertension | 21  4 | 57%  88% | 0.12 |
| (+) Diabetes  (-) Diabetes | 5  20 | 80%  58% | 0.36 |
| (+) Cardiovascular disease  (-) Cardiovascular disease | 8  17 | 75%  56% | 0.35 |
| (+) Obesity  (-) Obesity | 11  14 | 50%  71% | 0.22 |

^a^Calculated by Fisher’s Exact Test

**Table S3** Accuracy of CEUS in patients with purely solid lesions and cystic lesions or lesions with a cystic component^a^.

|  | **Solid Lesions (N=18)**  **Combined Readers** | | **Cystic Lesions (N=26)**  **Combined Readers** | |
| --- | --- | --- | --- | --- |
|  | Tissue diagnosis  (n=30) | Tissue or follow-up imaging  (n=36) | Tissue diagnosis  (n=16) | Tissue or follow-up imaging  (n=52) |
| Sensitivity | 100%  [28/28] | 100%  [28/28] | 86%  [12/14] | 86%  [12/14] |
| Specificity | 0%  [0/2] | 0%  [0/8] | 0%  [0/2] | 58%  [22/38] |
| Overall Accuracy | 93%  [28/30] | 78%  [28/36] | 75%  [12/16] | 65%  [34/52] |

^a^Results presented are based on combined readers and thus total number of diagnoses are twice the number of lesions.

**Table S4** Accuracy of CEUS compared to tissue diagnosis or follow-up imaging in patients with lesions <3 cm and $\geq$3 cm in patients with and without CKD^a,b^.

|  | **Smaller (<3 cm) Lesions (N=23)**  **Combined Readers** | | **Larger (**$\boldsymbol{\geq}$**3 cm) Lesions (N=21)**  **Combined Readers** | |
| --- | --- | --- | --- | --- |
|  | No CKD  (n=7) | CKD  (n=16) | No CKD  (n=12) | CKD  (n=9) |
| Sensitivity | 100%  (100%, 100%) | 75%  (32%, 95%) | 96%  (76%, 99%) | 100%  (100%, 100%) |
| Specificity | 0%  (0%, 0%) | 54%  (30%, 76%) | 0%  (0%, 0%) | 58%  (31%, 81%) |
| Overall Accuracy | 71%  (33%, 93%) | 56%  (34%, 76%) | 96%  (76%, 99%) | 72%  (46%, 89%) |

^a^For combined reader result, generalized estimating equations were used considering two readers' values as repeated measurements (or responses).

^b^Results presented as accuracy metrics (95% CI).
