# Supplementary material for: Diagnostic accuracy of contrast-enhanced ultrasound for characterization of kidney lesions in patients with and without chronic kidney disease
Source: BMC Nephrol. 2017 Aug 9;18:266. doi: 10.1186/s12882-017-0681-8 (PMC5551034; doi:10.1186/s12882-017-0681-8)
Supplement: Supplementary file 3 — Imaging parameters. (DOCX 13 kb) [file 12882_2017_681_MOESM3_ESM.docx]

**IMAGING PARAMETERS**

**CEUS**

CEUS was performed with the Siemens Acuson Sequoia 512 (Siemens, Mountain View, CA, USA) with contrast specific software using a 4C1 transducer. A standard MI of 1.9 was used for B-mode imaging and a low MI of 0.19 was used for all CEUS clips. Cadence pulse sequencing method was used for CEUS imaging. The center frequency for CEUS was 1.5 MHz, with a frame-rate of 14 Hz. The dynamic range was 80 dB, and the CEUS gain was manually set to minimize tissue signal prior to contrast injection. The range of gains used throughout the study was generally -10 to 0 dB.

The dose of contrast agent (Definity) was drawn up based on patient weight (0.5 mL for <125 lbs., 0.65 mL for 125-185 lbs., or 1 mL for >185 lbs.), diluted in normal saline (0.9%) to a final volume of 5 mL, and then bolus injected over 10 seconds through a minimum 20-gauge IV, followed by 10 mL normal saline (0.9%). A 3-minute clip was recorded commencing immediately prior to contrast injection.

**CT**

Contrast-enhanced CTs were acquired with a 16- to 64-slice MDCT scanner. A non-contrasted scan was first obtained. 100mL of Iohexol 755 mg/ml (Omnipaque 350 – GE Healthcare, Milwaukee, WI) was administered intravenously at 3-4mL/sec. Images were reconstructed in coronal and axial planes at 30s (arterial phase) and 100s (nephrographic phase) post contrast. The following parameters were used: kV 120, mAs 220, pitch 0.9, 5mm x 1.2 collimator for pre-contrast and nephrographic phase, 5mm x 0.6 collimator for arterial phase. Reconstructions: 5x3mm coronal thin MIPS of arterial phase, 5mm axials of all three phases.

**MRI**

MRs were performed on 1.5 Avanto and 3T Trio (Siemens, Iselin, NJ, USA). 10ml of Gadobenate Diglumine (Multihance, Bracco Diagnostics, Monroe Township, NJ) was administered intravenously. The protocol consisted of T1 FLASH (fast low angle shot) coronal, FLASH axial in and out of phase, T2 HASTE (half-Fourier acquisition single-shot turbo spin-echo) axial and coronal with fat saturation, T1 VIBE (volumetric interpolated breath-hold examination). Post contrast sequences were performed with VIBE fat saturation coronal and axial sequences, immediately, one and two minutes after injection.
